# DeWitt County Medical Society

**Published:** 1873-05-15

**Authors:** C. Goodbrake

**Affiliations:** Sec’y.


### DeWITT COUNTY MEDICAL SOCIETY.

The DeWitt County Medical Society met in annual session on the 29th day of April, 1873.

Members present—Drs. J. H. Potter, T. W. Davis, J. H. Tyler, J. Wright, Z. H. Madden, T. K. Edmiston, and Christopher Goodbrake. Also present—Dr. J. J. Starkey, Waynesville, and Dr. W. H. Crothers, Maroa, Macon county.

Dr. Potter, President, in the chair.

Minutes of last meeting read and approved.

On motion of Dr. J. Wright Dr. W. H. Crothers was elected an honorary member of the Society.

Dr. Wright proposed Dr. J. J. Starkey for membership; and the censors having reported favorably in his case, he was, on motion of Dr. Goodbrake, elected a member of the Society.

Election of officers being in order, the following gentlemen were elected for the ensuing year:

President, Dr. J. H. Tyler, DeWitt; Vice-President, Dr. T. W. Davis, Wapella; Secretary, Dr. C. Goodbrake, Clinton; Treasurer, Dr. Z. H. Madden, Clinton; Censors, Dr. W. G. Cochran, Farmer City; Dr. J. H. Potter, Wapella; Dr. J. J. Starkey, Waynesville.

Dr. Tyler, on taking the chair, delivered a short address, which was well received by the members present.

Dr. Thomas W. Davis reported the case of a gentleman in whom very strange symptoms occurred from the administration of an enema consisting of a tablespronful of common table salt in solution.

Dr. Tyler reported three very interesting cases of Cerebro-Spinal Meningitis.

Diseases of the Liver was chosen as the subject for discussion at the next meeting.

The essayists having failed to perform their duties, they were, by order of the President, continued.

On motion of Dr. Goodbrake, the following resolution was unanimously adopted:

*Resolved*, That the thanks of the Society are due and are hereby tendered to the editors of the *Clinton Public* and *Clinton Register* for kindly publishing notices of meetings of this Society.

On motion of Dr. Wright, the thanks of the Society were tendered to the proprietors of DeWitt Hall for inviting the Society to meet in their splendid room without any pecuniary remuneration.

On motion, the Secretary was ordered to furnish copies of the proceedings of this meeting, for publication in the Chicago Medical journals.

On motion of Dr. Madden, the Society adjourned to meet in quarterly session in Clinton, on the first Tuesday of July next.

C. GOODBRAKE, M. D. *Sec'y.*

